# The Unfolded Protein Response as a Modulator of Cancer‐Immune Cell Communication in the Tumour Microenvironment

**DOI:** 10.1111/jcmm.71380

**Published:** 2026-10-08

**Authors:** Yew Hwang Chee, Matthieu Moncan, Eileen Reidy, Adrienne M. Gorman, Afshin Samali

**Affiliations:** ^1^ Apoptosis Research Centre, School of Biological and Chemical Sciences University of Galway Galway Ireland; ^2^ School of Biological and Chemical Sciences University of Galway Galway Ireland

**Keywords:** antigen presentation, anti‐tumour immunity, cancer therapy, endoplasmic reticulum (ER) stress, immune modulation, secretome, tumour microenvironment (TME)

## Abstract

The tumour microenvironment (TME) comprises a dynamic network of structural, cellular, and signalling components that interact to influence tumour development. Immune cells play a key role in recognizing and eliminating cancer cells through detection of tumour‐derived membrane and secreted proteins processed in the endoplasmic reticulum (ER). These ER‐matured proteins on target cells act as critical communication signals, coordinating immune responses against tumour cells. However, during tumorigenesis, accumulation of unfolded or misfolded proteins in the ER of the cancer cells triggers the unfolded protein response (UPR), a transcriptional program that alleviates proteotoxic stress by reshaping the cancer cell proteome and secretome, thereby promoting survival. Activation of UPR disrupts intercellular communication and ultimately impairs immune‐mediated tumour clearance. Understanding this crosstalk could inform the development of therapeutics targeting UPR‐driven immune modulation. This review discusses how UPR‐signalling modulates tumour‐immune cell interactions within the TME.

## 1 Introduction

Tumour cells exhibit heterogeneous genetic alterations and dysregulation of key regulatory processes that affect molecular pathways controlling the cell cycle, signalling, metabolism, and survival [1]. These aberrations drive uncontrolled proliferation and resistance to cell death, establishing the foundation for tumour initiation and progression [1]. As tumours develop, tumour cells exploit these molecular changes to strengthen their ability to suppress or evade the immune response, for instance by expressing immune checkpoint molecules, such as programmed death‐ligand 1 (PD‐L1), a widely targeted molecule in tumour immunotherapy [2]. In parallel, tumour cells can release immunosuppressive cytokines that further dampen immune responses, presenting emerging targets for immune checkpoint/cytokine‐based therapies currently under clinical investigation [3].

Effective anti‐tumour immunity relies on the coordinated actions of innate and adaptive immune cells. Innate immune populations, including antigen‐presenting cells (APCs) and natural killer (NK) cells, provide the first line of immune defence by recognizing stress signals expressed on tumour cells [4]. Activated APCs, such as macrophages and dendritic cells (DCs), phagocytose tumour cells and secrete chemokines that recruit and prime adaptive T cells [4]. NK cells contribute to innate immunity by directly killing tumour cells through perforin‐ and granzyme‐containing cytolytic granules, and also by secreting cytokines and chemokines, such as interferon gamma (IFN‐γ), tumour necrosis factor‐alpha (TNF‐α), and C‐C motif chemokine ligand (CCL) 3–5, which enhance DC maturation/activation and promote T cell differentiation into effector and memory populations [5, 6]. Within the adaptive arm, T cells express T cell receptors (TCRs) that recognize tumour antigens presented by APCs via major histocompatibility complex (MHC) Class I or II molecules, or by tumour cells through MHC Class I [7]. Antigen recognition triggers clonal expansion, generating effector T cells with identical TCRs that precisely target tumour antigens and execute cytotoxic functions similar to NK cells [7]. However, TCR engagement alone is not sufficient to fully activate T cells. Additional signals from co‐stimulatory molecules such as CD28 are required to drive proliferation and differentiation [7]. While co‐stimulatory signals promote full T cell activation, these responses are counterbalanced by co‐inhibitory signals that can limit T cell function and prevent overactivation. Sustained engagement of co‐inhibitory receptors, including PD‐1 with its complementary ligand on tumour cells, contributes to T cell exhaustion, a state in which T cells progressively lose their proliferative capacity and effector functions [7]. Similarly, NK cell activity is governed by the balance between activating and inhibitory signals, which tumour cells can manipulate to suppress cytotoxic function and limit the elimination of tumour cells, thereby promoting evasion from anti‐tumour immunity [5]. Apart from granule‐mediated killing, both T and NK cells can induce tumour cell death by expressing death ligands such as Fas ligand or TNF‐related apoptosis‐inducing ligand (TRAIL), which engage TNF superfamily death receptors and/or calreticulin, an endoplasmic reticulum (ER) chaperone, on tumour cells to trigger apoptotic signalling [8, 9]. When operating effectively, this coordinated network maintains control over tumour growth and prevents tumour progression.

Tumour malignancy is further reinforced by the tumour microenvironment (TME), a complex network comprising tumour vasculature, stromal cells such as tumour‐associated fibroblasts, infiltrating immune cells, and a repertoire of soluble signalling molecules [1]. Within this environment, tumour cells and components of the TME can shape immune responses through paracrine signalling, including the secretion of immunosuppressive cytokines like interleukin (IL)‐6 and IL‐10, which reprogram infiltrating immune cells toward tumour‐supportive phenotypes [10]. In addition, tumour‐derived chemokines further recruit immunosuppressive populations, such as myeloid‐derived suppressor cells, tumour‐associated macrophages, tumour‐associated neutrophils, and regulatory T cells, all strongly associated with immune resistance and poor clinical outcomes [11]. Collectively, these TME‐driven immunosuppressive mechanisms, produced by tumour cells and recruited immune populations, reinforce the suppressive network, promoting tumour progression and enabling immune evasion [11].

Central to this intercellular communication is the secretory pathway, which relies on the ER and Golgi apparatus for the production, maturation, and trafficking of membrane‐bound proteins (e.g., death ligands and receptors) and secreted signalling proteins such as chemokines and cytokines [12]. Proper ER function is therefore essential for effective crosstalk between tumour and immune cells. In tumours, however, oncogenic stress, including genotoxic, oxidative, and metabolic challenges can disrupt ER homeostasis, leading to the accumulation of unfolded or misfolded proteins, a condition known as ER stress [12].

ER stress is sensed by three transmembrane ER proteins: inositol‐requiring enzyme 1α (IRE1α), protein kinase RNA‐like ER kinase (PERK), and activating transcription factor 6 (ATF6) [12]. Upon activation, IRE1α, a dual kinase and ribonuclease, mediates unconventional splicing of X‐box binding protein 1 (XBP1) mRNA to produce the transcription factor XBP1s, which drives expression of chaperones and ER folding enzymes [12]. In parallel, IRE1α also leads to the degradation of selective mRNAs and microRNAs via regulated IRE1‐dependent decay (RIDD), thereby reducing the ER protein load and alleviating stress [12]. PERK responds to ER stress by phosphorylating eukaryotic translation initiation factor 2α (eIF2α), which transiently inhibits global protein translation, thereby preventing further accumulation of unfolded proteins [12]. At the same time, it allows the translation of activating transcription factor 4 (ATF4), which upregulates genes involved in protein folding, redox balance and metabolic adaptation, enabling the cell to cope with stress [12]. Meanwhile, ATF6 is transported to the Golgi upon ER stress, where it is proteolytically cleaved to release a cytosolic transcription factor [12]. This transcription factor induces genes that improve protein folding and enhance the clearance of misfolded proteins [12].

Together, these three sensors initiate the unfolded protein response (UPR), an adaptive signalling network that restores ER proteostasis or triggers apoptosis under unresolvable stress [12]. Beyond maintaining ER function, the UPR also modulates tumour‐relevant processes such as metabolic reprogramming, sustained proliferation, and resistance to cell death [13, 14, 15]. Emerging evidence indicates that UPR signalling also reshapes the TME and regulates immune responses, yet its role in tumour‐immune cell communication remains underexplored. In this review, we explore current research on how the UPR influences crosstalk between tumour and immune cells, emphasizing mechanistic insights from diverse cancer models. We primarily focus on UPR signalling originating within tumour cells and how it shapes their interaction with the immune system. However, UPR activation can also occur directly within the infiltrating immune cells in response to conditions in the TME, providing an additional layer of regulation of immune cell function.

## 2 Role of the UPR in Shaping Tumour Immune Evasion

Anti‐tumour immunity relies on three critical steps: tumour recognition by the immune system, activation of tumour‐killing immune cells, and recruitment of these cells to the tumour site [16]. Each step requires precise signalling between tumour and immune cells through receptors and secreted factors, which tumour cells often disrupt to evade the immune system.

### 2.1 UPR Regulates Immune Recognition of Tumour Cells

Adaptive immune cells detect tumour cells through antigen presentation, a process in which cytosolic proteins are degraded by the proteasome into peptides [17]. During early tumorigenesis, mutations in oncogenes or tumour suppressors generate aberrant proteins that are processed into neoantigens and presented on MHC‐I molecules [7]. This increased neoantigen presentation enhances tumour visibility to immune cells [18]. After proteasomal degradation, peptides are translocated into the ER lumen with the help of transporter associated with antigen processing (TAP), loaded onto MHC‐I complexes, and stabilized by chaperones such as Tapasin before trafficking to the cell surface [17]. Whereas normal cells present self‐peptides that are tolerated by CD8^+^ cytotoxic T cells, tumour cells present tumour‐specific antigens that can be recognized by TCRs on CD8^+^ T cells (Figure 1) [17]. While CD8^+^ T cells recognize peptides presented by MHC‐I, NK cells monitor MHC‐I expression itself [17]. They are inhibited by normal levels of MHC‐I, allowing them to spare healthy cells, but become activated when MHC‐I is downregulated or when tumour‐specific antigens are presentated on stressed or tumour cells [17]. MHC‐I expression suppresses NK cells primarily through inhibitory killer‐cell immunoglobulin‐like receptors (KIRs) and NK Group 2 member A (NKG2A) signalling [17].

**FIGURE 1:**
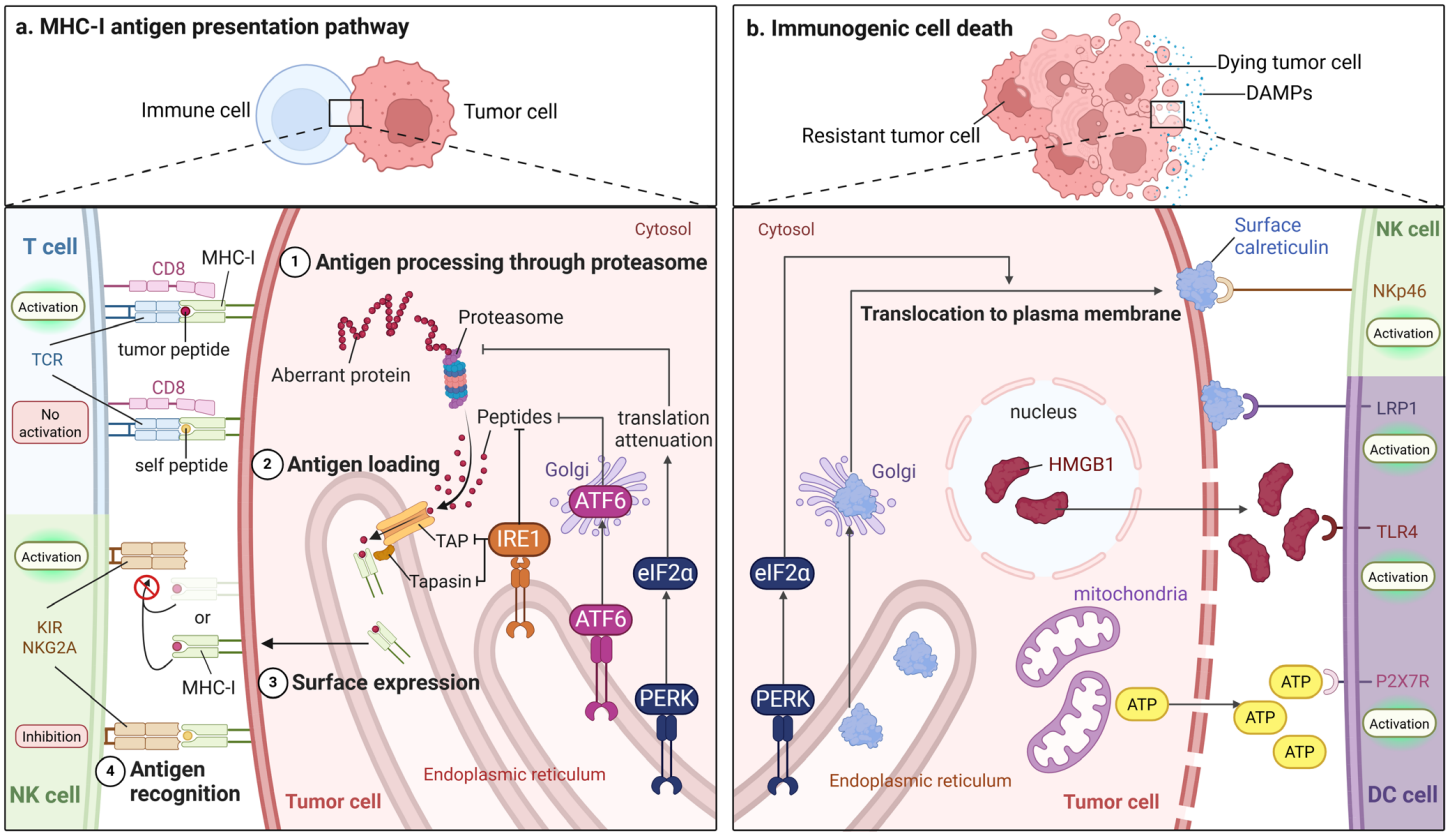
The UPR regulates immune recognition through antigen presentation and immunogenic cell death (ICD) pathways. (a) In the MHC‐I antigen presentation pathway, (1) proteasome‐generated peptides, including self‐derived peptides and tumour‐derived aberrant peptides are produced in the cytosol. These peptides are transported into the ER by the transporter associated with antigen processing (TAP). (2) They are loaded onto MHC‐I complexes with the help of chaperones, such as Tapasin. (3) Peptide–MHC‐I complexes are then trafficked to the cell surface for presentation. (4) Both CD8^+^ T cells and NK cells recognize tumour cells through changes in MHC‐I presentation, but via distinct mechanisms. A detailed explanation of the mechanisms shown is provided in the main text. Following TCR engagement with antigen‐MHC complexes, a second co‐stimulatory signal is required for full T cell activation. In tumour cells, the MHC‐I antigen presentation pathway can be modulated by UPR signalling. IRE1α signalling can impair TAP/Tapasin expression and promote degradation of antigenic peptides, thereby decreasing MHC‐I surface expression and facilitating immune evasion. ATF6 activation has also been reported to suppress MHC‐I surface presentation, although the underlying mechanisms remain unclear. PERK signalling may limit antigen presentation by reducing peptide availability through eIF2α‐mediated translational attenuation. (b) During ICD, PERK‐eIF2α signalling promotes surface exposure of calreticulin, which acts as an ‘eat‐me’ signal recognized by NK cells through NKp46 and DC through LRP1 respectively, thus promoting activation of NK and DC cells and anti‐tumour immune responses. Dying tumour cells also release DAMPs, such as ATP and HMGB1, which are normally retained intracellularly. Extracellular ATP and HMGB1 are recognized by P2X7R and TLR4, respectively, promoting immune cell activation. (Created in BioRender. Chee, Y. H. (2026) https://BioRender.com/zr2lr1g).

As MHC molecules are not essential for cell survival, IRE1α signalling can contribute to immune evasion by downregulating MHC‐I surface expression. In human embryonic kidney cells, expression of UPR‐activating human homeostatic iron regulator (HFE) C282Y mutant reduces surface MHC‐I without altering total protein levels, an effect that is reversed by XBP1s silencing [19]. Consistently, in human lung and cervical cancer cells, ER stress induced by tunicamycin through inhibition of N‐linked glycosylation increases XBP1s, which upregulates miR‐346 [20]. This microRNA targets TAP1 mRNA and reduces TAP1 protein, thereby impairing peptide translocation into the ER [20]. XBP1 inhibition also restores surface MHC‐I proteins in tongue squamous cell carcinoma by suppressing tripeptidyl peptidase 2 (TPP2), a protease that degrades antigenic peptides [21]. Since XBP1 functions downstream of ATF6, silencing ATF6 similarly restores surface MHC‐I levels in the HFE C282Y model, supporting the idea that antigen presentation is regulated by multiple UPR branches [19]. Moreover, IRE1‐RIDD selectively degrades MHC‐I heavy chains and *Tapbp* mRNAs in mouse DCs, reducing surface MHC‐I levels and limiting CD8^+^ T cell activation, highlighting that UPR‐mediated regulation of antigen presentation occurs across different cell types [22, 23]. Collectively, these findings demonstrate that IRE1α signalling can suppress MHC‐I antigen presentation at multiple stages of the antigen‐processing pathway, thereby reducing tumour visibility to cytotoxic immune cells and contributing to immune evasion. Importantly, the regulation of antigen presentation is not restricted to IRE1α, as other UPR branches can also influence the availability of antigenic peptides for MHC‐I presentation. Consistently, PERK signalling dampens antigen presentation in murine lymphoid tumour cells. PERK‐mediated eIF2α phosphorylation decreases global protein synthesis and likely limits peptide availability, resulting in reduced MHC‐I presentation [24, 25].

Another way the immune system detects tumour cells is through immunogenic cell death (ICD), a form of cell death characterized by changes in cell surface composition and the release of immunogenic soluble factors from the dying cells [26, 27]. These factors, known as damage‐associated molecular patterns (DAMPs), act as ‘danger’ signals recognized by immune cells through specific DAMP receptors, thereby triggering an immune response [26, 27]. In tumours, exposure to immunogenic chemotherapy or radiation causes the release of DAMPs such as calreticulin, high mobility group box 1 (HMGB1) and ATP, which are normally retained inside the cells [27]. During the early course of ICD, calreticulin translocation to the plasma membrane serves as an ‘eat me’ signal for DCs via low density lipoprotein receptor‐related protein 1 (LRP1), thereby promoting phagocytosis of dying cells, leading to DC maturation, and enhanced antigen presentation to T cells [27, 28].

Importantly, not all anti‐tumour treatments induce ICD equally. Different therapies vary in their ability to generate ER stress or ROS required for robust ICD activation [29]. Although all three UPR branches may activate during ICD, PERK has been identified as a major UPR sensor required for this process, as PERK‐eIF2α signalling mediates calreticulin exposure in response to multiple chemotherapeutics and UV irradiation [29, 30]. PERK is thought to initiate caspase‐8‐mediated signalling that promotes calreticulin translocation from the ER to the cell surface via the Golgi apparatus [29]. Supporting this, the ablation of general control nonderepressible 2 (GCN2), another eIF2α kinase, also reduces eIF2α phosphorylation and mitoxantrone‐induced DAMPs release [30]. Inhibition of PERK or GCN2 in murine prostate tumour cells reduces DC activation in vivo [30]. PERK‐dependent ICD not only promotes DC activation but also facilitates NK cell recognition. NK cells can recognize surface‐exposed calreticulin via the activating receptor NKp46 and in murine melanoma, doxorubicin‐induced ER stress promotes calreticulin exposure and the interaction of calreticulin with NKp46 on NK cells enhances doxorubicin's anti‐tumour effect [31]. Interestingly, ICD can also occur when PERK is inhibited in ER‐stressed murine melanoma cells, with dying cells exposing calreticulin and releasing HMGB1 and ATP to drive anti‐tumour immunity independent of caspase activation [32]. In addition, the ER chaperone binding immunoglobulin protein (BiP) that maintains the three UPR sensors in an inactive state under normal conditions could also contribute to ICD [12]. Inhibition of BiP enhances ionizing radiation‐induced ICD in human glioma stem cells (GSCs) through activation of PERK and IRE1α [33]. BiP silencing increases surface calreticulin exposure and the extracellular release of ATP and HMGB1 [33]. Phagocytosis of these ICD‐induced GSCs promotes DC maturation and T cell activation [33]. However, the observation that ICD can occur despite PERK inhibition indicates that PERK is an important, but not universally required, mediator of ICD. This suggests that the requirement for PERK may depend on the type of cellular stress and the tumour cell type. In some contexts, other UPR pathways, including IRE1α, may contribute to the induction of ICD [33].

### 2.2 UPR Disrupts Immune‐Mediated Tumour Cell Death

After initial antigen recognition, T cells require additional co‐signalling receptors to determine the outcome of TCR signalling, ensuring appropriate commitment to activation or tolerance through co‐stimulatory and co‐inhibitory signals (Figure 2) [34]. PD‐L1 is a key co‐inhibitory molecule expressed by many tumour cells that binds the PD‐1 receptor on T cells to suppress T cell activation and promote immune evasion [34]. The UPR can modulate expression of co‐inhibitory signals, thereby limiting anti‐tumour immunity. Treatment with ER stressors such as thapsigargin or HA15, a BiP inhibitor, induces PD‐L1 expression in human melanoma cells through IRE1α RNase activity [35]. In addition, the ER chaperone BiP has been proposed to directly regulate PD‐L1 protein stability, as thapsigargin increases PD‐L1 protein levels in triple negative breast cancer, head and neck cancer and pancreatic adenocarcinoma cell lines [36]. Importantly, combining HA15 with anti‐PD‐1 therapy enhances CD8^+^ T cell infiltration and cytotoxic activity in murine melanoma models, indicating that UPR‐mediated modulation of immune checkpoints can be therapeutically exploited [35].

**FIGURE 2:**
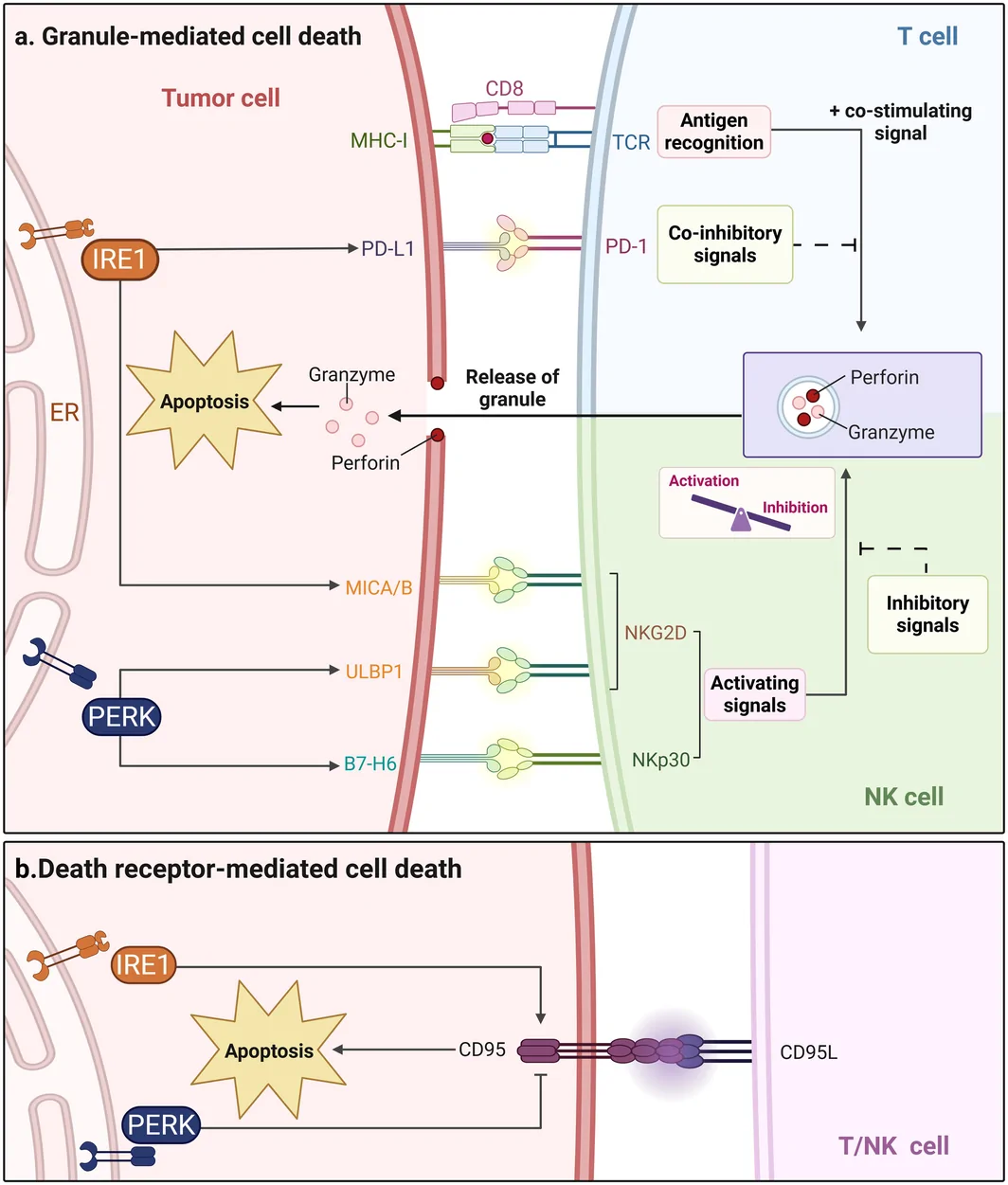
The UPR disrupts immune‐mediated tumour cell death. Cytotoxic T cells and NK cells eliminate target cells through two major mechanisms. (a) Release of cytotoxic granules containing perforin and granzymes. In T cells (upper right), antigen recognition alone is insufficient for full activation and requires co‐stimulatory signalling to achieve complete activation, whereas co‐inhibitory pathways suppress T cell activation despite antigen engagement. One key co‐inhibitory pathway involves PD‐1 binding to PD‐L1 on tumour cells. In NK cells (lower right), activation is governed by the balance between activating and inhibitory signals. NK cells can be activated through receptors such as NKG2D, which recognize stress‐induced ligands on tumour cells, including MICA/B and ULBP1, as well as NKp30, which binds B7‐H6. However, these activating signals can be opposed by inhibitory signals, with the net balance determining NK cell activation. Perforin within the granules can form pores in the target cell membrane, enabling granzymes to enter the cell and trigger apoptotic cell death. (b) Target cells can also be eliminated through death receptor signalling, such as CD95‐mediated apoptotic pathways. The UPR modulates immune activation and cytotoxicity by regulating the expression of immune checkpoints, stress‐induced ligands, and death receptor pathways in tumour cells. Specifically, IRE1α has been reported to regulate PD‐L1, MICA/B, and CD95 expression, whereas PERK regulates ULBP1, B7‐H6, and CD95. (Created in BioRender. Chee, Y. H. (2026) https://BioRender.com/166axi1).

In addition to regulating T cell responses, the UPR also influences NK cell cytotoxicity. Apart from inhibitory receptors such as NKG2A discussed above, NK cell activation is determined by a balance between inhibitory and activating receptor signals [5]. NK cells can detect stressed or transformed cells through activating receptors such as NK Group 2 member D (NKG2D) [37]. NKG2D recognizes stress‐induced ligands, including the MHC Class I polypeptide‐related sequence A and B (MICA/B), which are normally expressed at low levels but are upregulated in transformed or stressed cells, thereby acting as ‘kill me’ signals [37]. However, UPR signalling can suppress this axis. IRE1α‐XBP1s signalling represses MICA expression in human melanoma cells via inhibition of E2F transcription factor 1 (E2F1), a transcriptional activator of MICA, whereas ER stress induced by tunicamycin reduces MICA/B expression post‐transcriptionally in human hepatocellular carcinoma cells [38, 39]. Consistently, UPR‐mediated inhibition of MICA/B reduces NK‐mediated cytotoxicity [38]. The UPR, through PERK‐eIF2α‐ATF4 signalling, also regulates additional NK‐activating ligands, including UL16 binding protein 1 (ULBP1) and B7‐H6, ligands for NKG2D and NK natural cytotoxicity receptors 3 (NKp30), respectively, which in turn influence NK cell cytotoxic responses [40, 41, 42].

Beyond modulation of activating and inhibitory ligands, tumour cells can also be eliminated through the extrinsic death receptor pathway [9, 43]. In this pathway, death receptors expressed on tumour cells engage their cognate ligands on immune cells, initiating caspase‐dependent cell death [9, 43]. Accordingly, the Fas receptor (Aka CD95) is differentially regulated by the two arms of IRE1α signalling, with XBP1s promoting CD95 expression while RIDD suppresses it [44]. Moreover, PERK knockdown alters CD95 protein levels under ER stress without affecting IRE1α signalling, highlighting coordinated regulation of death receptor expression by multiple UPR branches [44].

These findings suggest that the immunological consequences of tumour intrinsic UPR are not determined by a single immune‐regulatory molecule, but by context‐dependent changes in the tumour cell surface landscape that can differentially influence T cell and NK cell responses.

### 2.3 UPR Interferes With Tumour‐Immune Communication

Apart from direct cell–cell contact, tumour‐immune communication also relies on cytokines, chemokines, and extracellular vesicles (EVs) (Figure 3) [45]. Cytokines are soluble proteins that transmit signals between cells, while chemokines specifically regulate the migration and activation of phagocytes and lymphocytes, acting in a context‐dependent manner to either enhance or suppress immune responses, which can be modulated by ER stress [46]. ER stress induced by thapsigargin in murine prostate cancer, metastatic melanoma and Lewis lung carcinoma cells promotes the release of soluble factors including cytokines IL‐6, IL‐23 and TNF‐α [47]. Conditioned medium from these cells activates ER stress signalling in macrophages, promoting their polarization toward a pro‐inflammatory phenotype, partly via Toll‐like receptor (TLR4), a receptor that detects DAMPs [47]. In addition, these transmissible ER stress signals can simultaneously impair DC cross‐presentation, thereby limiting CD8^+^ T cell activation in vitro and infiltration in vivo [48]. By contrast, HA15 induced ER stress activates XBP1s/RIDD signalling in human melanoma cells and enhances the secretion of chemokines C‐X‐C motif ligands (CXCL) 9–11, as well as IL‐6, and TNF‐α [35]. This shift in the tumour secretion increases infiltration of cytotoxic CD8^+^ T cells into the TME in vivo [35]. When combined with anti‐PD‐1 antibody treatment, HA15 further enhances this effect [35]. In TNBC, basal and paclitaxel‐enhanced IRE1α activity modulates the tumour secretome by promoting pro‐inflammatory factors such as IL‐6, IL‐8, CXCL1, granulocyte‐macrophage colony‐stimulating factor (GM‐CSF) and transforming growth factor‐beta 2 (TGFβ2) [49]. Likewise, IRE1α activity in glioblastoma cells regulates the recruitment of myeloid cells [50]. In vivo, ablation of IRE1α significantly reduced the infiltration of polymorphonuclear neutrophils into the tumour core and limited the accumulation of microglia/macrophages at the tumour periphery [50]. Interestingly, beyond cytokines and chemokines, tumour‐intrinsic UPR signalling can also influence immune cell composition through other secreted mediators. In murine non‐small cell lung cancer, loss of tumour IRE1α increases DC infiltration and enhances CD4^+^ and CD8^+^ T cell effector activity, partly through reduced production of the immunosuppressive lipid mediator prostaglandin E2 (PGE_2_) [51].

**FIGURE 3:**
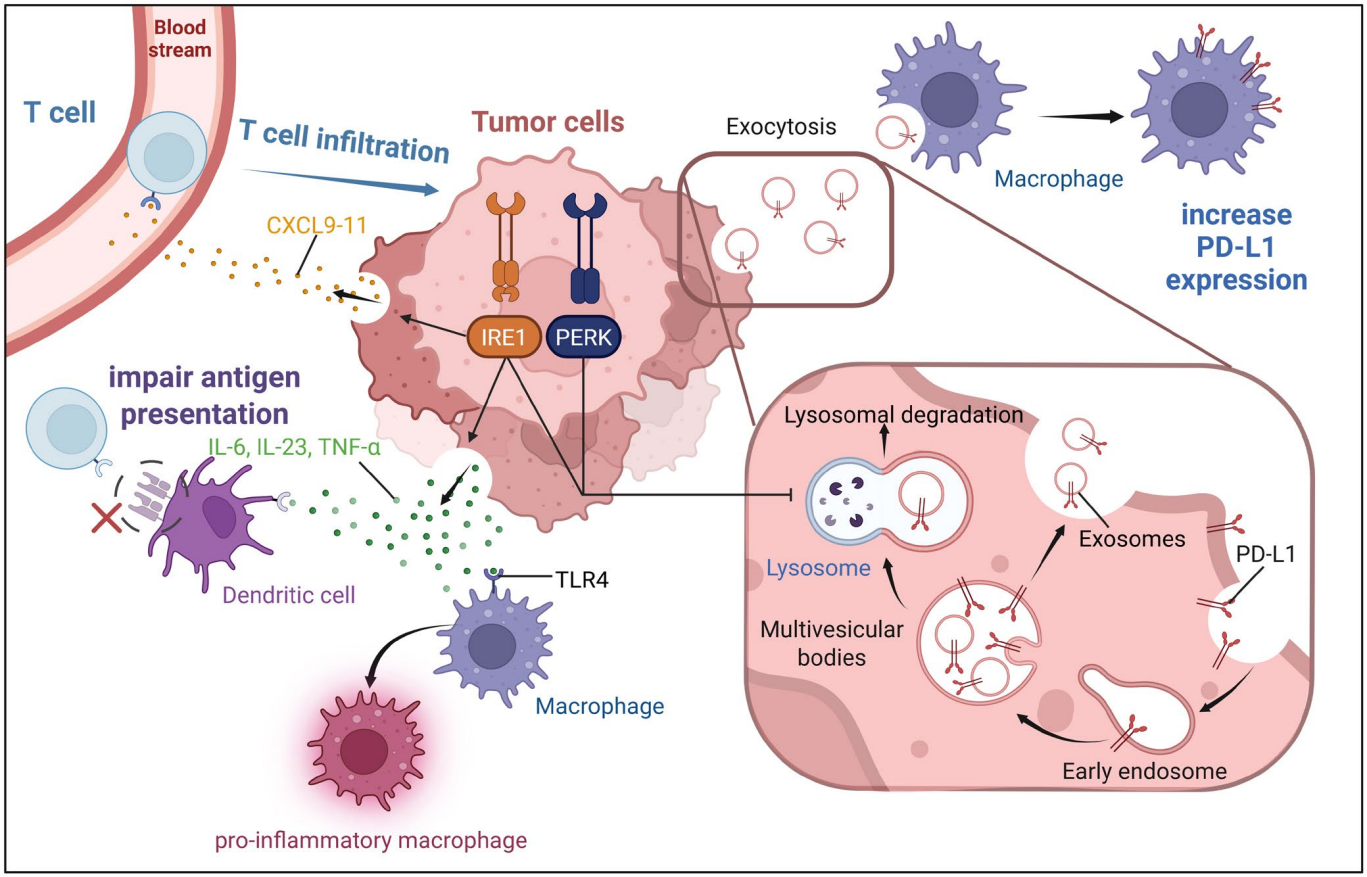
The UPR interferes with tumour‐immune cell communication. Tumour cells release chemokines such as CXCL‐9/10/11 to promote immune cell recruitment, including increased infiltration of cytotoxic CD8^+^ T cells into the tumour microenvironment. Cytokines such as IL‐6, IL‐23 and TNF‐α released by tumour cells can regulate the activation of immune cells in a context‐dependent manner. They can reduce dendritic cell cross‐presentation to T cells while simultaneously inducing the polarisation of macrophages to a pro‐inflammatory phenotype via TLR4 signalling. Tumour cell‐derived exosomes can carry functional cargo, including donor cell‐derived PD‐L1, which modulates recipient immune cell behaviour, including the upregulation of PD‐L1 expression in macrophages. Exosomes are formed within multivesicular bodies (MVBs), which originate from early endosomes (bottom right). MVBs can either fuse with lysosomes for cargo degradation or undergo exocytosis, releasing exosomes into the extracellular space. IRE1α has been demonstrated to regulate the expression of cytokines and chemokines, while both PERK and IRE1α can influence MVB formation. (Created in BioRender. *Chee, Y. H*. (2026) https://BioRender.com/xldcogc).

Exosomes are membrane‐bounded extracellular vesicles formed from multivesicular bodies (MVBs), which fuse with the plasma membrane to release their contents into the extracellular space via exocytosis, representing another mode of immune modulation [52]. Tumour‐derived exosomes act as vital information carriers, as they can transport functional cargo like mRNAs, miRNAs, lipids, membrane‐bound proteins or cytosolic proteins from tumour cells to recipient immune cells, where they can induce functional reprogramming [52]. PERK and IRE1α kinase activities have been shown to regulate the biosynthesis of MVBs and exosome production in tumours [53]. Indeed, under thapsigargin‐induced ER stress, IRE1α and PERK promote exosome secretion by limiting lysosomal degradation of MVBs [54]. Mechanistically, ATF4 reduces lysosome acidification by inhibiting vesicular‐ATPase assembly, which normally pumps protons into lysosomes to maintain the acidic environment required for cargo degradation [54]. Furthermore, ER stress promotes the release of exosomes containing PD‐L1 or specific miRNAs such as miR‐27a‐3p [55, 56]. Uptake of PD‐L1‐containing exosomes by macrophages promotes their polarization toward a pro‐tumourigenic M2 phenotype in vivo [56]. Similarly, co‐culture of macrophages with tumour‐derived exosomes enriched in miR‐27a‐3p upregulates macrophage PD‐L1 expression both in vitro and in vivo [55]. Severe ER stress in BeWo choriocarcinoma cells further enhances release of EVs carrying pro‐inflammatory DAMPs, reinforcing the idea that UPR facilitates the transmission of pathological signals through diverse EVs in tumours [57].

Importantly, the effects of UPR signalling on tumour‐immune communication are not restricted to changes originating in tumour cells. Immune cells within the TME can themselves undergo UPR activation in response to tumour‐derived factors and environmental stress, altering their metabolic and functional state. In glioblastoma, tumour‐derived factors activate PERK‐ATF4 signalling in monocyte‐derived macrophages, promoting metabolic adaptations including increased GLUT1 expression, glucose uptake and lactate production, which support the immunosuppressive macrophage phenotype [58]. Similarly, in melanoma, PERK‐ATF4 signalling in macrophages promotes serine biosynthesis, mitochondrial respiration and lipid oxidation through the ATF4‐PSAT1 pathway [59]. Genetic or pharmacological inhibition of PERK reduces the immunosuppressive activity of M2 macrophages and enhances anti‐tumour T cell responses in a murine melanoma model [58]. PERK signalling also regulates myeloid‐derived suppressor cell function through nuclear factor erythroid 2‐related factor 2 (NRF2), with PERK loss promoting mitochondrial dysfunction and STING‐dependent immune activation, thereby reducing myeloid‐derived suppressor cell‐mediated immunosuppression and enhancing CD8^+^ T cell responses in Lewis lung carcinoma and melanoma models [60]. Together, these findings show that UPR signalling can interfere with tumour‐immune communication at multiple levels. Tumour‐intrinsic UPR signalling alters the soluble and vesicular signals delivered to immune cells, while UPR activation within immune cells determines how they respond to the TME.

## 3 Therapeutic Targeting of the UPR and Its Effects on Tumour Immunity

Given the central role of the UPR in modulating tumour‐immune cell communication, targeting its pathways represents a promising strategy to enhance cancer therapies.

Inhibition of IRE1α can reshape the TME, influencing immune cell infiltration and anti‐tumour activity. Treatment with an IRE1α kinase inhibitor (Compound 18) significantly reduced the abundance of tumour‐associated fibroblasts and myeloid‐derived suppressor cells in breast tumour xenografts, likely via transcriptional downregulation of cyclooxygenase 2 (COX‐2), CXCL8, and CXCR4 [61]. In Myc‐driven 2153 L genetically engineered mouse mammary tumour cells transplanted into BALB/c mice, inhibition of IRE1α RNase with MKC8866 combined with docetaxel enhanced CD4^+^ and CD8^+^ T cell infiltration while reducing myeloid‐derived suppressor cells compared to single treatments [62]. Similarly, MKC8866 synergized with anti‐PD‐1 to increase infiltration of CD8^+^ T and NK cells into the TME of a murine prostate cancer model [63]. MKC8866 is currently in Phase 2 clinical trials (ORIN1001, NCT03950570) [64].

PERK inhibition also modulates tumour immunity. Deletion of PERK in melanoma cells reduced tumour growth in wild‐type mice but not in Rag1‐deficient mice that lack functional T cells, indicating the anti‐tumour effect is immune‐dependent [32]. Similarly, PERK ablation combined with CD4^+^ or CD8^+^ T cell blockade confirmed its role in T cell regulation [32]. In PERK knockout tumours, CD8^+^ T cells exhibited elevated PD‐1 expression, and anti‐PD‐1 therapy further enhanced tumour suppression [32]. Early studies in C57BL/6 mice with MB49 bladder cancer cells suggest that PERK inhibition using HC‐5404 modestly reduces tumour growth and enhances anti‐PD‐1 efficacy by increasing CD8^+^ T cell infiltration and activation [65]. Whether these effects arise from tumour‐intrinsic PERK inhibition or its impact on immune cells remains to be fully clarified. HC‐5404 is currently being evaluated in a Phase 1 trial in multiple solid tumours, including renal cell carcinoma, gastric cancer, metastatic breast cancer, and small cell lung cancer (NCT04834778) [66].

Collectively, these studies indicate that UPR inhibition can reshape tumour‐immune interactions, facilitating recognition and elimination of tumour cells and enhancing responses to immunotherapies. By restoring proper communication between tumour and immune cells, UPR modulation may overcome tumour‐induced immune evasion and improve therapeutic outcomes.

## 4 Future Perspectives

Cellular crosstalk within the TME is a critical determinant of anti‐tumour immunity. Tumours are often classified as ‘cold’ or ‘hot’ based on immune cell infiltration [16]. Cold tumours exhibit low immune infiltration and reduced activation signals, making them less responsive to immunotherapies such as immune checkpoint inhibitors. In contrast, hot tumours, with high immune infiltration, are generally more responsive to immunotherapy [16]. Strategies that convert cold tumours into hot tumours by enhancing infiltration of activated immune cells are therefore of great interest.

The UPR is increasingly recognized as a key regulator of tumour immune landscapes. By modulating the expression of membrane‐bound and secreted proteins, UPR activation shapes the tumour secretome and surface antigen presentation, thereby altering how tumour cells are recognized by immune cells and how they communicate with the surrounding TME. However, the effects of UPR activation on tumour immunity represent a double‐edged sword. Acute ER stress can promote tumour immunogenicity and immune‐mediated tumour cell elimination, whereas chronic ER stress can support tumour adaptation, immune evasion and suppression of anti‐tumour responses. The outcome of UPR activation therefore depends on factors such as the UPR pathway engaged, the intensity and duration of ER stress, tumour lineage and the surrounding immune cells. Understanding these factors will be important for developing therapies that modulate the UPR to promote anti‐tumour immunity without compromising the protective functions of the stress response.

Therapeutic targeting of UPR pathways may therefore provide one approach to restore proper tumour‐immune communication and enhance the efficacy of immunotherapies. For example, in TNBC, co‐treatment with docetaxel and the IRE1α inhibitor MKC8866 converts the immunologically cold tumours into hot tumours, thereby restoring sensitivity to immune checkpoint inhibitors [67]. However, when targeting the UPR, one must consider its essential role in maintaining ER proteostasis in both tumour and immune cells. Inhibition of UPR pathways may impair the function or survival of highly secretory or ER stress‐sensitive immune cells, potentially limiting the therapeutic window. Careful selection of UPR targets, dosing strategies and combination with immunotherapy may help maximize anti‐tumour effects while preserving the normal function of immune cells.

Several questions remain regarding UPR‐targeted strategies in cancer therapy. Can restoring tumour‐immune cell communication enhance responses to cell‐based therapies, such as CAR‐T or CAR‐NK cells? Given that UPR activation promotes immune suppression through inhibitory receptor expression, it may limit the efficacy of such approaches. Additionally, which tumour types and contexts benefit most from UPR modulation? Cancers with high levels of basal UPR activation might be particularly responsive. Interestingly, UPR induction can simultaneously impair DC cross‐presentation while enhancing pre‐activated CD8^+^ T cell function in co‐culture experiments [35, 48]. This suggests that UPR modulation may be most effective in tumours with pre‐existing immune infiltration [16].

Optimising UPR‐targeted interventions will require identifying effective therapeutic combinations and reliable biomarkers to guide patient selection. Importantly, because the UPR is essential for immune cell survival and function, targeting it may have unintended effects on immune populations, despite potential benefits for anti‐tumour immunity [68]. Indeed, UPR modulation is already being investigated in chronic inflammatory and autoimmune diseases, further highlighting its importance in immune‐linked conditions.

## Author Contributions

**Yew Hwang Chee:** writing – original draft, visualization, writing – review and editing. **Matthieu Moncan:** writing – original draft, writing – review and editing. **Eileen Reidy:** visualization, writing – review and editing. **Adrienne M. Gorman:** visualization, funding acquisition, writing – review and editing. **Afshin Samali:** conceptualization, funding acquisition, writing – review and editing.

## Funding

This work was supported by Taighde Éireann‐Research Ireland, 23/SPP/12064, 13/RC/2073_P2. Taighde Éireann‐Research Ireland Government of Ireland Postgraduate Scholarship, GOIPG/2022/1708.

## Conflicts of Interest

Afshin Samali and Adrienne M. Gorman are co‐founders and directors of Cell Stress Discoveries Ltd. Yew Hwang Chee, Matthieu Moncan and Eileen Reidy declare no conflicts of interest.

## Data Availability

Data sharing not applicable to this article as no datasets were generated or analysed during the current study.
